# Therapeutic targeting of FLT3 and associated drug resistance in acute myeloid leukemia

**DOI:** 10.1186/s13045-020-00992-1

**Published:** 2020-11-19

**Authors:** Melat T. Gebru, Hong-Gang Wang

**Affiliations:** 1 Department of Pediatrics, Pennsylvania State University College of Medicine, Hershey, PA USA; 2 Department of Pharmacology, Pennsylvania State University College of Medicine, Hershey, PA USA; 3 Penn State College of Medicine, 500 University Drive, Hershey, PA 17033 USA

**Keywords:** AML, FLT3, Drug resistance, Drug tolerance

## Abstract

Acute myeloid leukemia (AML) is a heterogeneous disease caused by several gene mutations and cytogenetic abnormalities affecting differentiation and proliferation of myeloid lineage cells. FLT3 is a receptor tyrosine kinase commonly overexpressed or mutated, and its mutations are associated with poor prognosis in AML. Although aggressive chemotherapy often followed by hematopoietic stem cell transplant is the current standard of care, the recent approval of FLT3-targeted drugs is revolutionizing AML treatment that had remained unchanged since the 1970s. However, despite the dramatic clinical response to targeted agents, such as FLT3 inhibitors, remission is almost invariably short-lived and ensued by relapse and drug resistance. Hence, there is an urgent need to understand the molecular mechanisms driving drug resistance in order to prevent relapse. In this review, we discuss FLT3 as a target and highlight current understanding of FLT3 inhibitor resistance.

## Introduction

Acute myeloid leukemia (AML) is a hematological malignancy that is characterized by a rapid clonal expansion of abnormally differentiated myeloid progenitor cells (blasts) [1]. Overall, the 5-year survival rate of AML patients, based on data collected from 2009 to 2015, is 28.3% [2]. The prognosis and survival of AML patients are highly dependent on various factors, mainly the mutation profile and age of the patient. While patients under the age of 60 have a 40–50% survival probability, those over the age of 60 have a much worse prognosis with only 10–20% survival [1]. This is partially attributed to the fact that older patient population has a higher proportion of patients with unfavorable mutation profile and their inability to tolerate intensive chemotherapy [3]. AML arises from a series of genetic alterations of hematopoietic stem cells accrued with age or caused by prior therapy, such as radiation or treatment with topoisomerase II inhibitors or alkylating agents [3].

AML is a heterogeneous disease caused by an array of genetic changes, but two co-occurring genetic events are crucial for leukemogenesis: class I mutations that activate signal transduction pathways leading to proliferation and class II mutations that alter transcription factors involved in myeloid differentiation [4, 5]. Transcription factors required for differentiation are commonly disrupted by chromosomal translocations that result in fusion proteins that act as dominant negative form of the wild-type protein, such as RUNX1-MTG8, CBFβ-MYH11, MLL-AF6/9 and PML-RARα [4]. The prognostic impact of chromosomal translocations is highly variable. Depending on the affected gene and the function of the fusion protein, the outcome for patients could range from favorable to adverse risk with high to low probability of survival.

About 50% of AML patients do not have cytogenetic/chromosomal abnormalities [6]. Recent advances in genomics have uncovered specific gene mutations or changes in gene expression in AML that are now used to predict prognosis and guide treatment. The most recurrently mutated genes in AML include nucleoplasmin 1 (*NPM1*), Fms-like tyrosine kinase 3 (*FLT3*), DNA methyltransferase 3A (*DNMT3A*), isocitrate dehydrogenase (*IDH1 and IDH2*) and ten–eleven translocation 2 (*TeT2*) mutations [7]. Mutations in DNMT3A, IDH 1 and 2, and TeT2 affect DNA methylation and contribute to leukemogenesis through epigenetic modifications of hematopoietic stem cells affecting their development and differentiation. However, in order to become malignant, leukemic clones not only need to evade the tight regulation of differentiation through chromosomal translocations and mutations of epigenetic modifiers, but also need to acquire mutations that induce unrestrained proliferation. Mutations in the receptor tyrosine kinases (RTKs) FLT3 and KIT as well as the Ras family of oncogenes provide proliferative advantage for pre-leukemic clones and account for two-thirds of all AML mutations [8]. These mutations rarely overlap, and that is possibly because of the redundancy of their function [7].

FLT3 is one of the most sought-out therapeutic target due to the fact that it is frequently overexpressed or mutated, and its mutations are associated with poor prognosis in AML. There has been a sustained effort to develop FLT3 inhibitors leading to approval of two drugs and several others in advanced clinical trials. Although patients initially respond very well to FLT3 inhibitors, the clinical duration of response is often short-lived as patients relapse with more aggressive and drug-resistant disease [9, 10]. The exact mechanism of resistance to FLT3 inhibitors remains elusive. Here, we review the biology of FLT3 and discuss different mechanisms of FLT3 inhibitor resistance as well as the initial stages of drug resistance preceding an overt relapse.

## FLT3

### FLT3 structure and biology

**Fig. 1:**
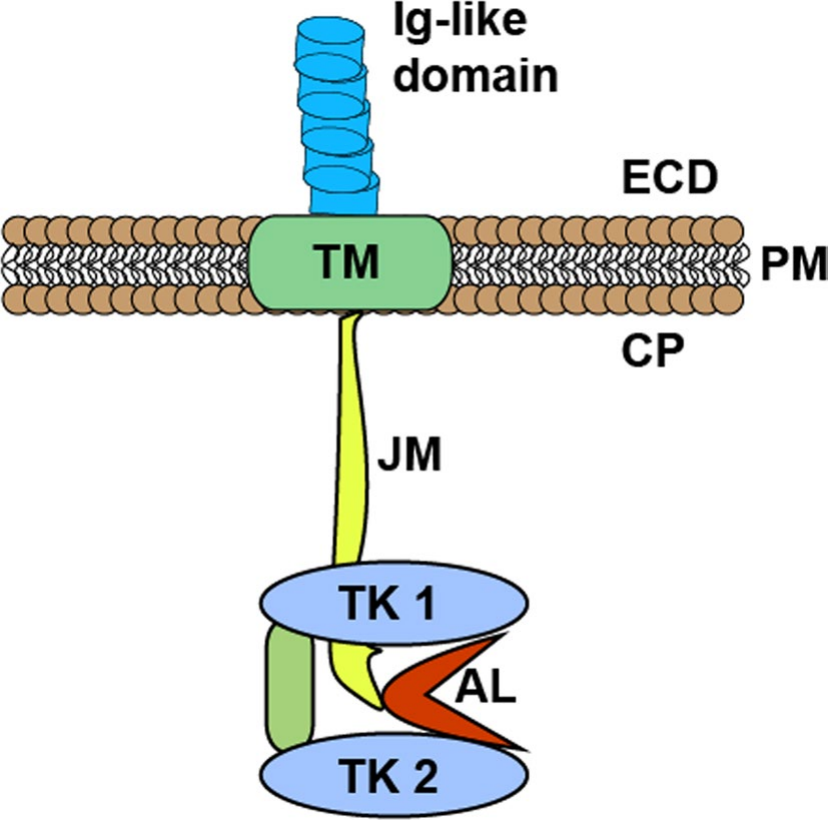
Schematics illustrating a monomeric FLT3. Glycosylated FLT3 is anchored on the plasma membrane (PM) with the transmembrane domain (TM), and its immunoglobulin-like ligand binding domain protrudes out to the extracellular domain (ECD). In the cytoplasm (CP), the juxtamembrane domain (JM) extends and connects with the two kinase domain lobes (TK1 and TK2) that are linked by the activation loop (AL)

FLT3 belongs to class III family of RTKs and shares homology with other members of the family, such as the PDGFR (platelet-derived growth factor receptor), KIT (stem cell factor receptor) and M-CSF (macrophage colony stimulating factor) [11]. Structurally, FLT3 comprises four regions (Fig. 1): (1) an N-terminal extracellular region consisting of five immunoglobulin-like subdomains, (2) a transmembrane domain, (3) a juxtamembrane (JM) domain, and (4) an intracellular C-terminal kinase domain consisting of two substructures (N-lobe and C-lobe) that are connected by an activation-loop (A-loop) [11–13]. The extracellular region of FLT3 is glycosylated and contains a ligand binding domain as well as a dimerization domain [12, 13]. The nonglycosylated form of the receptor is not anchored to the plasma membrane [13]. The JM domain plays an important regulatory role through direct contact with the catalytic kinase domain [14]. Finally, the kinase domain transmits activation signal to downstream targets and is regulated by the conformation of the A-loop and the JM domain as well as ATP binding [12, 13, 15].

In normal hematopoiesis, FLT3 is selectively expressed on CD34 + hematopoietic stem cells and myeloid as well as B-lymphoid progenitors, but is absent in erythroid progenitors [16, 17]. Although FLT3 is predominately expressed in hematopoietic tissues, it could also be detected in the brain, testis and placenta [13, 17]. On the other hand, FLT3 ligand (FL) is expressed ubiquitously except in the brain, suggesting that the expression pattern of the receptor is the limiting factor in determining tissue specificity [18–20]. FL is a type 1 transmembrane protein that could be either alternatively spliced or proteolyzed to yield a soluble form of the ligand. FLT3 can be activated by a secreted or membrane-bound FL through a paracrine or autocrine feedback loop [18–20].

### FLT3 activation and signal transduction

Wild-type FLT3 (WT-FLT3) is monomeric when inactive, and binding of its ligand, FL, induces receptor dimerization [12, 13]. Once activated, the now dimeric receptor bound to FL is internalized and degraded within 20 min. The auto-inhibitory activity of the JM domain mediates a steric inhibition causing the receptor to remain in inactive conformation [14]. Hence, a rapid self-regulation and receptor internalization play a key role in instituting a negative feedback control.

FL-mediated dimerization and activation of FLT3 induce auto-phosphorylation of tyrosine residues on the receptor [13]. The A-loop, a flexible peptide that folds between the split kinase domains (N- and C-lobes), contains tyrosine residues that can be auto-phosphorylated [14]. When the receptor is inactive and the A-loop is not phosphorylated, it folds between the N- and C-lobes and blocks the binding of ATP and substrates. When the receptor is activated, the A-loop remains in an open conformation allowing the binding of ATP and activation of the kinase [13, 14]. Additionally, the conformation of the A-loop is regulated by the conformation of the JM domain which is also phosphorylated during receptor activation [14].

Upon FL binding, auto-phosphorylation of the tyrosine residues in the receptor creates docking sites for downstream adapter proteins with Src homology 2 (SH2) domains, such as GRB2 (growth factor receptor-bound protein 2) and SHC (Src homology 2 containing protein), inducing multiple signaling cascades [21, 22]. WT-FLT3 mainly signals through Ras/MAPK (mitogen-activated protein kinase) and PI3K/Akt (phosphatidylinositol-3-kinase/protein kinase B) pathways [12, 13].

The Ras/MAPK pathway culminates in activation of ERK1/2 (extracellular signal regulated kinase, also known as MAPK) [23]. ERK1/2 plays a key role in executing the upstream FLT3 activation and growth signal by phosphorylating and activating multiple transcription factors involved in proliferation, including CREB (cyclic adenosine monophosphate response element-binding protein), c-Myc and AP-1 [13, 24]. ERK also induces the expression of negative regulators of the pathway, such as Spry (sprouty) and DUSP (dual specificity phosphatases) family of proteins [25].

FLT3-derived activation of the PI3K/Akt pathway results in phosphorylation of mTOR (mechanistic target of rapamycin) [24, 26], which increases overall protein synthesis and regulates several genes involved in proliferation and survival through inhibition of 4E-BP1 (eukaryotic transcription initiation factor 4E-binding protein) and activation of p70S6Kinase (p70S6K) [13, 27]. Furthermore, Akt can also block apoptosis by inhibiting the anti-apoptotic protein Mcl-1 degradation and phosphorylating the pro-apoptotic protein BAD [12, 13, 27].

The activation of the Ras/ERK and PI3K/Akt pathways often occurs in parallel with phosphorylate many common targets involved in survival as well as cell cycle regulation, including cyclins, CDKs (cyclin-dependent kinases), checkpoint kinases and negative regulators of cell cycle, like p27^Kip1^, that is blocked by activated FLT3 [28, 29].

### FLT3 mutations in AML

WT-FLT3 is overexpressed in 93% of AML cases as well as in 80–90% of B- and T-ALL (acute lymphoblastic leukemia) [11, 30]. Furthermore, FLT3 is the most commonly mutated gene in AML with mutations observed in approximately 30% of all AML cases and 70% of patients with normal karyotype [11]. There are two major types of FLT3 mutations: (i) internal tandem duplication (ITD) frequently in the JM domain of the receptor observed in about 25% of AML patients and (ii) point mutations in the tyrosine kinase domain (TKD mutations) in about 7% of cases [11, 13].

### FLT3-ITD mutation

A seminal discovery of FLT3-ITD mutation in 1996 by Nakao et al. [31] established the importance of FLT3 in AML. In AML, ITD often occurs in exons 14 and 15 of FLT3 (coding for the JM domain) with duplication of different bases, ranging from 3 to over 400, in multiples of three while maintaining the reading frame [32]. The cause of FLT3–ITD mutation is not completely clear. One possible mechanism proposed includes a DNA replication error caused by the palindromic sequence found in the region where duplication often occurs, which is the tyrosine-rich region of the JM domain (codon 589–599). In this scenario, the ITD occurs due to a subsequent impaired DNA mismatch repair [33].

The ITD mutation causes ligand-independent constitutive receptor dimerization and auto-phosphorylation resulting in receptor activation [12, 32]. This activation is caused by disruption of the JM domain’s inhibitory activity through a conformational change that prevents its association with the kinase domain. In WT- FLT3, in order for the JM domain to relieve its inhibitory effect on the A-loop, it requires a ligand-dependent auto-phosphorylation of tyrosine residues in the JM region [34]. Interestingly, the added tyrosine residues in the JM domain due to duplication have been demonstrated to have no role in constitutive activation of the receptor [32].

### FLT3-ITD signaling pathways

Although FLT3-ITD mutant activates the same downstream targets as WT-FLT3, including PI3K/Akt and Ras/MAPK, it can also uniquely activate STAT5 [12, 13, 32]. It has been shown that the tyrosine residues Y589 and Y591 in the JM domain get exposed by ITD allowing for possible docking of SH2 domain of STAT5 and triggering the STAT5 pathway [35, 36]. Replacing the tyrosine residues with phenylalanine was shown to abrogate STAT5 phosphorylation and activation. This is further supported by a study that demonstrated that inhibition of JAK2 or TYK2 or Src family kinases did not block STAT5 activation in FLT3-ITD cells [35, 36]. It has also been reported that FLT3-ITD mutation affects its glycosylation and localization of the receptor [37]. The nonglycosylated FLT3-ITD is partially retained in the ER-Golgi network, and the ER-localized receptor is not only exempt from endocytic degradation, but also could signal via STAT5 more efficiently than the plasma membrane-anchored receptor [38].

Activated STAT5 dimerizes and translocates into the nucleus where it induces transcription of multiple targets involved in cell proliferation and survival, including cyclin D1, c-Myc, p21, and PIM (proviral integration site for Moloney murine leukemia virus) serine-threonine kinases (PIM-1 and PIM-2) [35, 36]. The PIM family of kinases is involved in a number of oncogenic pathways in various cancers, especially in myeloma and leukemia. PIM directly phosphorylates serine residues of Cdc25A, c-Myc, and Notch-1, inducing their activation and promoting proliferation [39, 40]. On the other hand, PIM-induced phosphorylation of the CDK inhibitors p21^Cip1/Waf1^ and p27^kip1^ as well as the pro-apoptotic protein BAD results in their inactivation contributing to cell cycle progression and blockade of apoptosis [39, 40]. Interestingly, it has been shown that PIM-1 can directly phosphorylate FLT3 on its serine residue, S935, resulting in the stabilization and ER-retention of the nonglycosylated 130 kDa form of the receptor [38, 40]. This in turn promotes the activation of STAT5 and increases the expression of PIM-1 resulting in a positive feedback loop [40]. Inhibition of PIM-1 results in decreased STAT5 activation and PIM-1 expression [40]. Moreover, the co-inhibition of FLT3-ITD and PIM-1 has been shown to synergize in inducing apoptosis, making it a promising target in AML [40].

Furthermore, a previous study has shown that FLT3-ITD is associated with increased ROS (reactive oxygen species) production as compared to WT-FLT3 due to the fact that FLT3-ITD can activate STAT5 [32]. Activated STAT5 binds to and activates Rac-1 which plays an important role in ROS production [41]. Even a partial knockdown of STAT5 was shown to decrease ROS production. Importantly, the increase in ROS was also shown to be associated with increased genomic instability due to increased DNA double-strand breaks (DSBs) repair through nonhomologous end joining (NHEJ) [41].

### FLT3 point mutations

The second most common type of FLT3 mutation in AML is point mutation within the tyrosine kinase domain (TKD) [32]. Although TKD mutations may occur independent of ITD mutations, they can sometimes be detected together either on the same or opposite allele [42]. The most common TKD mutations occur within the A-loop of TKD2, mainly involving the aspartic acid (D835), isoleucine (I836) and tyrosine (Y842) residues. Mutations within TKD1 like N676 and F691 are also observed albeit to a lesser extent [32].

The A-loop blocks access of ATP and substrates to the kinase domain when the receptor is in an inactive state [13]. Point mutations that result in substitution of these important amino acid residues affect the inhibitory effect of the A-loop leading to constitutive kinase activation and signaling through the Ras/MAPK and PI3K/Akt pathways [43].

The prognostic significance of FLT3-TKD mutations is not as clear as FLT3-ITD mutations [43, 44]. Some studies have found weak impact of TKD mutations on prognosis [44, 45], whereas others have found no association [43]. It is interesting that while both FLT3-ITD and TKD mutations ultimately lead to constitutive activation of the receptor and downstream signaling, they have markedly different impact on prognosis. Although the exact mechanism is unknown, it is possible that the alternative signaling through STAT5 in FLT3-ITD clones contributes to the aggressiveness of the disease. It is also possible that ER-anchored FLT3-ITD can interact with other cytosolic proteins that augment cell proliferation and survival pathways.

### FLT3-targeted inhibitors

FLT3 is one of the most important targets in AML, and there has been a sustained effort to develop FLT3 inhibitors since the discovery of FLT3 mutations. FLT3 inhibitors are small molecules that compete with ATP to bind the active pocket of the kinase domain, inhibiting auto-phosphorylation and phosphorylation of downstream targets [46]. FLT3 inhibitors can broadly be categorized into first- and second-generation inhibitors. The first-generation FLT3 inhibitors are multikinase inhibitors and thus not selective to FLT3; some examples include midostaurin, sorafenib, sunitinib, and ponatinib (Table 1) [46, 47]. The second-generation FLT3 inhibitors are developed to selectively inhibit FLT3 and include quizartinib, gilteritinib, and crenolanib (Table 1) [46, 47].

**Table 1:** Summary of FLT3 inhibitors and mechanisms of resistance observed

Inhibitor name		Generation	Type	Known targets	Clinical development phase	Observed mechanism of resistance
Midostaurin		First	I	FLT3, PKC, KIT, PDGFR, VEGF, CDK1, etc	Approved for newly diagnosed FLT3 mutant AML patients in combination with chemotherapy	Upregulation of MCL-1 [61, 62], FLT3-N676K mutation [63]
Sorafenib		First	II	FLT3, VEGFR, KIT, RET, PDGFR RAF-1, ERK	Phase III (NCT02156297), used off-label	FLT3-D835Y, F691L, Y842D mutations [44, 48, 64, 65], increased PIM expression [66], ERK activation [67]
Sunitinib		First	I	PDGFR, KIT, VEGFR, RET	Phase I and II (NCT00783653)	Loss of FLT3 [68]
Ponatinib		First	II	FLT3, BCR-ABL, VEGFR	Phase I/II (NCT02428543), (NCT02829840)	FLT3-D835Y mutation [69]
Quizartinib		Second	II	FLT3, KIT, PDGFR	Phase III (NCT02668653)	FLT3-D835Y, F691L, Y842D mutations [48, 70], ERK activation [67]
Gilteritinib		Second	I	FLT3, AXL	Approved for FLT3 mutant adult AML patients who failed or are refractory to previous treatment	FLT3-F691L mutation, N-Ras signaling [71]
Crenolanib		Second	I	FLT3, PDGFR	Phase III (NCT03258931)	N-Ras, IDH2, TET2 [72], FLT3-K429E, F691L [69, 73]

FLT3 inhibitors can also be categorized into type I and II inhibitors based on how they bind to FLT3. Three conserved residues, aspartate–phenylalanine–glycine (DFG), in the A-loop of the kinase domain of FLT3 flip to attain either a ‘DFG-in’ or ‘DFG-out’ conformation when FLT3 is active or inactive, respectively [46]. Type I FLT3 inhibitors, such as midostaurin, crenolanib, and gilteritinib, bind to the ATP binding pocket only when the receptor is active (DFG-in). On the other hand, type II inhibitors, including quizartinib and sorafenib, interact with the hydrophobic region adjacent to the ATP binding domain which can only be accessed when the receptor is inactive (DFG-out) [46]. The most common FLT3-TKD mutation at residue D835 typically replaces the negatively charged aspartate with a hydrophobic amino acid, disrupting the inactive conformation [48]. Hence, type II inhibitors can only block FLT3-ITD, but not TKD mutant receptors, while type I inhibitors can block both. Another common mutation at the residue F691 in the kinase domain mutates the hydrophobic pocket and is associated with conferring drug resistance [48]. Midostaurin and gilteritinib have recently been approved by the US FDA and are detailed below. Several other FLT3 inhibitors are in advanced clinical trials and are summarized in Table 1.

#### Midostaurin

Midostaurin (PKC412) is the first drug to be approved by the US FDA for the treatment of FLT3 mutant AML [49, 50]. Midostaurin is a staurosporine derivative initially found to inhibit protein kinase C (PKC) [51] but later found to have activity against several other kinases, including KIT, PDGFR, VEGF, CDK1, FLT3, etc. [52]. Anti-FLT3 activity of midostaurin was discovered in an apoptosis screen using Ba/F3 cells expressing FLT3-ITD [53]. Midostaurin is a type I FLT3 inhibitor and was found to inhibit auto-phosphorylation and downstream signaling of FLT3-ITD [54]. In a phase I clinical trial, 75 mg midostaurin given twice daily was found to be well tolerated with no adverse side effects [55]. After oral administration, midostaurin is rapidly absorbed with a maximum plasma level (C_max_) reached within 1–1.5 h and binds to alpha-1 acidic glycoprotein (AAG) in the plasma [55]. Midostaurin is metabolized in the liver and has two major metabolites, CGP 52421 and CGP 62221, which showed an extended half-life of 36 h [55]. In a phase II study, single agent midostaurin showed a transient reduction in peripheral blast count by over 50% in FLT3 mutant AML patients who were refractory and/or relapsed (R/R) after a prior chemotherapy [54]. However, midostaurin had no significant effect on bone marrow blast cells [54]. Hence, the subsequent phase III trial was limited to testing it in combination with other treatments. The phase III trial, “RATIFY”, recruited from 17 countries a total of 717 newly diagnosed AML patients with either FLT3-ITD or -TKD mutations aged 18–60 years [56]. Patients were stratified by FLT3-TKD, ITD high (> 0.7) or low (< 0.7) allelic ratio. The treatment arm received 50 mg of midostaurin twice daily for 13 days subsequent to both induction and consolidation chemotherapy and as post-treatment maintenance for twelve 28-day cycles [56]. The placebo group received standard chemotherapy plus placebo. Although the complete remission (CR) rate in the midostaurin arm was not significantly higher than the placebo arm (59% vs. 54%), the 5-year survival rate was significantly higher in the midostaurin-treated group as compared to placebo (50.8% vs. 26.7%) [56]. Midostaurin benefited FLT3-ITD high and low as well as FLT3-TKD patients similarly [56], suggesting that at least some of its efficacy could be attributed to its activity against other kinases besides FLT3. Based on data from RATIFY, FDA granted a breakthrough status to midostaurin in 2016 and later approved it for treatment of newly diagnosed FLT3 mutant AML patients in combination with chemotherapy in 2017 [49, 50].

#### Gilteritinib

Gilteritinib (ASP2215) is a highly selective type I FLT3 inhibitor with activity against the tyrosine kinase Axl, which has been shown to be involved in FLT3 inhibitor resistance [57]. Gilteritinib is the second FLT3 inhibitor to be approved by the FDA for the treatment of FLT3 mutant AML. In preclinical experiments, gilteritinib was shown to inhibit phosphorylation of FLT3 and its targets [57]. In phase I/II clinical trials, gilteritinib was well tolerated with a maximum tolerated dose of 300 mg/day and the most common treatment-related adverse events included diarrhea, fatigue, anemia, and elevation of liver enzyme [58]. A randomized, phase III trial (“ADMIRAL”) was conducted in 14 countries and recruited 371 adult AML patients with FLT3-ITD or -TKD mutations who were refractory to prior chemotherapy [59]. The gilteritinib group (247 patients) received 120 mg of gilteritinib daily for a 28-day cycle, and the control group received standard salvage chemotherapy of choice (high or low dose) [59]. Gilteritinib-treated patients had a significantly longer median overall survival than the chemotherapy group (9.3 vs. 5.6 months) [59]. There was no significant difference in the median overall survival between FLT3-ITD (9.3 months) and FLT3-TKD (8 months) mutant patients who received gilteritinib. The composite complete remission (CRc) rate was 34% in the gilteritinib group as compared to 15.3% in the chemotherapy group [59]. Furthermore, a higher percentage of patients was able to bridge to hematopoietic stem cell transplant (HCT) in the gilteritinib group (25%) as compared to chemotherapy group (15.3%) [59]. In 2018, gilteritinib was granted approval for the treatment of FLT3 mutant adult AML patients who failed or are refractory to previous treatment [60].

### Relapse and drug resistance in AML

Although 80% of AML patients achieve a complete remission after induction and consolidation therapy, most of them relapse and fewer than 30% of the patients survive over 5 years [74]. Similarly, AML patients treated with targeted therapy, such as IDH2 or FLT3 inhibitors, almost always relapse unless patients receive subsequent HCT [9, 10, 75, 76]. Relapse is caused by a small number of leukemic clones that are able to survive treatment and eventually reestablish often a more aggressive and drug-resistant leukemia.

Remission is commonly defined as < 5% blast cells in the bone marrow based on morphological analysis [74, 77]. However, the introduction of more sensitive techniques in recent years has enabled the detection of minimal or measurable residual disease (MRD). Techniques such as flow cytometry, RT-PCR and next-generation sequencing (NGS) can be used to detect MRD with varying degrees of sensitivity ranging from 10^–4^ to 10^–6^ [74]. Typically, MRD tests look for the presence of a specific mutation identified during diagnosis, although testing for different mutations could also be done [78]. Patients positive for MRD almost always relapse. A study that analyzed peripheral blood samples of patients at different time points after chemotherapy found that 92% of MRD-positive FLT3 mutant AML patients relapsed as compared to 35% MRD-negative patients [78]. The overall survival of all patients, regardless of mutation type, was significantly higher in MRD negative as compared to MRD-positive patients (73% vs. 24%, respectively) [78]. While MRD testing is not a part of the standard clinical practice, it is commonly performed after patients complete a treatment cycle, and the presence of MRD is used to determine the next treatment strategy. Although there is often a difference of a few months between molecular and hematological relapse, MRD-positive patients often undergo another round of chemotherapy or targeted therapy or HCT in a preemptive effort to completely eradicate the leukemic clones [74, 77].

FLT3 mutant clones are difficult to completely eradicate, and patients with FLT3 mutations are more likely to have MRD after chemotherapy and eventually relapse [78]. Similarly, relapse and resistance after undergoing treatment with FLT3-targeted inhibitors are almost inevitable. Understanding the mechanism of resistance to FLT3-targeted therapy is crucial to eradicate MRD-causing clones. The exact mechanism by which MRD clones survive and eventually lead to resistance is not completely clear. There are several factors that can influence response to therapy, which can be receptor and nonreceptor related.

Mutation of drug target is the most common receptor-intrinsic mechanism of resistance to targeted therapy. Indeed, this is commonly observed in patients treated with type II FLT3 inhibitors, such as quizartinib and sorafenib, which bind FLT3 only when the kinase is in the inactive DFG-out conformation. Patients treated with these drugs acquire point mutations in the kinase domain, often at D835 and F691 residues (Table 1), which cause constitutive activation of the kinase and block type II inhibitors from binding. Based on this clinical observation, drugs that can target both FLT3-ITD and -TKD mutations, including gilteritinib and crenolanib, were developed [48, 79]. However, resistance to those inhibitors can still develop through nonreceptor mechanisms that reactivate downstream targets [80]. Loss of the FLT3 receptor is another resistance mechanism observed in relapse patients. One study that analyzed the variant allele frequencies (VAF) of FLT3 mutation before and after crenolanib treatment found that 11 out of 21 FLT3-D835 mutant and 11 out 39 FLT3-ITD mutant patients completely lost their FLT3 VAF after treatment [72]. Similar loss of FLT3 has been reported by other studies [71, 81].

The bone marrow microenvironment (BM) has been implicated in mediating a receptor-independent mechanism of MRD clone survival by providing a sanctuary for leukemic clones that is difficult to access by drugs in the plasma. Infiltration of the BM is a key indicator of efficacy of a FLT3-targeted drug [82]. However, even drugs that are able to access the BM are not able to completely eradicate AML cells. Studies have shown that bone marrow stromal cells can interact with AML cells and regulate drug response and cell fate, including survival, proliferation, differentiation, and self-renewal [83]. A recent study cocultured FLT3 mutant AML cell lines with several proteins from the BM and found that FLT3 ligand and fibroblast growth factor 2 (FGF2) were able to confer resistance to quizartinib treatment [84]. They showed that FGF2 binds to FGF receptor (FGFR) on AML cells and reactivates the MAPK pathway driving survival and proliferation [84]. They tested the expression of FGF2 in bone marrow biopsies taken from FLT3-ITD AML patients before treatment, during response to quizartinib, and at relapse and found that FGF2 was significantly increased during relapse [84]. Several other BM factors such as IL-3, GM-CSF [85], CXCR4/CXCL12, VLA-4, E-selectin [83], and marrow CYP3A4 [86] have been implicated in driving resistance to therapy. In addition to promoting survival of AML cells, BM can drive differentiation of AML cells in response to treatment. It has been shown that while FLT3 inhibitors such as quizartinib and gilteritinib induce cell death in the peripheral blood and suspension culture in in vitro, they induce differentiation in the BM as well as in a coculture with bone marrow stromal cells [87, 88].

Other nonreceptor-related mechanism of MRD clone survival includes pharmacokinetic factors at the cellular level, such as decrease in drug uptake, increase in drug efflux, and inactivation of drug by intracellular metabolism. It has been shown that increased expression of the ABCB1 gene also known as P-glycoprotein (Pgp) or multidrug resistance protein 1 (MDR1), a member of the ABC transporter family that can efflux drugs out of the cell, is strongly associated with resistance to chemotherapy and FLT3 inhibitors in AML [89, 90].

Furthermore, MRD clone survival could be mediated through drug-induced genetic mutations and epigenetic modulations to alter gene expression in order to rewire signaling pathways to negate/compensate for the effect of FLT3 inhibition. This is commonly observed in patients treated with type I inhibitors that target both FLT3-ITD and -TKD mutations. Some of the resistance-conferring mutations observed in the clinic include mutations of N-Ras, K-Ras, B-Raf, PTPN11, Cbl, and IDH [71, 72], as well as upregulation of Bcl-2, Mcl-1, and KIT [62, 80, 91].

### Mechanisms of resistance development

A crucial question to ask, in order to prevent acquisition of resistance, is how these resistance mechanisms develop. Resistance-conferring mutations could be harbored in a small, initially undetectable, preexisting subclone selected during the drug treatment [92]. Alternatively, mutations can arise de novo due to drug pressure or in parallel with drug treatment, but independent from drug action [92].

Evidence supporting the preexisting resistant subclone hypothesis in AML is limited due to the technical difficulties of detecting such low-frequency subpopulations of cells. However, deep sequencing analyses have demonstrated that AML is heterogeneous consisting of multiple clones that exist at various levels of frequencies, which can change posttreatment. For instance, one study utilized deep sequencing to analyze AML samples pre- and posttreatment with crenolanib (a type I FLT3-ITD and TKD inhibitor) [72]. They found that low allele frequencies of N-Ras and IDH2 mutations were detected in pretreatment samples in separate subclones from FLT3 mutant clones, but were amplified in resistant posttreatment samples [72]. Another study that demonstrated that resistance-conferring mutations could be preexisting in small subclones used a xenograft model to expand cells from pre- and post-sorafenib treatment AML patient samples in NOD/SCID (immunodeficient) mice; they found that D835Y-positive clones were expanded which were only detected in the paired post-sorafenib treatment samples at relapse, but not during diagnosis [93]. More evidence supporting preexisting resistant-clone hypothesis is anticipated to emerge as single-cell sequencing technologies advance.

On the other hand, it is plausible that genomic instability imposed by therapy as well as intrinsic to the cancer cells could increase the frequency of de novo mutations that result in drug resistance. Two studies employed deep whole genome sequencing and single-nucleotide polymorphism array profiling for paired diagnosis and relapse patient samples and found that the relapse samples had acquired new genomic alterations undetected in the diagnosis samples [94, 95].

It is likely that both preexisting and acquired resistance-conferring mutations play a role in relapse. Interestingly, an elegant study using a BRAF mutant melanoma model demonstrated that treatment with BRAF inhibitors induces drug-sensitive cells to secrete factors that promote the survival, proliferation, and metastasis of preexisting resistant clones [96]. While the mechanism of expansion of preexisting clones is relatively simple, how drug-sensitive cells acquire resistance-conferring mutations is less clear. Particularly, understanding the mechanism by which drug-sensitive cells tolerate and survive treatment prior to transitioning to a fully resistant state is crucial to prevent resistance and relapse.

### Drug-tolerant persisters

Drug-tolerant persisters (DTPs) were first described by Sharma *et a*l. in 2010 [97]. They identified DTPs while testing the acute response of EGFR mutant nonsmall cell lung cancer cells (NSCLCs) to a lethal EGFR inhibition. They found that a small subpopulation of single clone-derived cells can survive treatment with lethal doses of EGFR inhibitor despite lacking resistance-conferring mutations [97]. These surviving DTPs were transiently quiescent and reversibly resistant to EGFR inhibition where, after culture in drug-free media, they resume proliferation and regain sensitivity to EGFR inhibitors [97]. They also demonstrated that DTPs alter their chromatin state, have “stem-like” phenotype, and maintain IGF1 signaling for survival [97]. Following this seminal paper, other studies have shown that across multiple solid tumors, DTPs survive targeted, as well as chemotherapeutic, treatment through a variety of mechanisms such as upregulation of lipid hydroperoxidase, micro-RNAs and various proliferative and anti-apoptotic signaling pathways [92, 98–100]. Furthermore, recent studies supported Sharma and colleagues’ findings in the same lung cancer model and further showed that when DTPs were exposed to EGFR inhibitors for an extended period of time, they developed permanent resistance-conferring genetic mutations [92, 100].

In AML, DTPs cause MRD which eventually leads to relapse and drug resistance. The exact mechanism of DTPs survival during MRD remains incompletely understood. Our recent study has demonstrated that FLT3 mutant AML cells can survive and tolerate lethal FLT3 inhibition despite lacking resistance-conferring mutation [101]. Leukemia stem cells (LSCs) have been implicated in driving drug tolerance and relapse, especially in the context of chemotherapy [102–104]. LSCs are defined as dormant subpopulation of cells with self-renewing capacity and resistance to chemotherapy and other anti-proliferative drugs [103]. A recent study assessed LSC, as defined by CD34 + CD38-cells, frequency in 869 AML patients at diagnosis and after achieving complete remission (CR) [105]. They found that LSC frequency can predict overall survival independently as well as in combination with MRD analysis [105]. Patients who were MRD^high^/LSC^high^ had the worst prognosis and highest relapse rate as compared to patients who were MRD^low^/LSC^low^ [105].

Although the transcriptional profile and surface marker expression of LSCs have been shown to be similar to hematopoietic stem cells (HSCs), LSCs have a plastic gene expression pattern that allows them to be in a dynamic state between stem- and nonstem-like cells [103, 106]. Studies using a variety of cancer models have demonstrated that cancer cells can revert to a stem-like state de novo in response to environmental stimuli [97, 106, 107]. A mathematical model has been proposed to describe this cell-state transition as a single-cell stochastic behavior to promote phenotypic equilibrium [108]. This can also explain why drug-resistant DTPs regenerate a drug-sensitive equilibrium state following exit from quiescence upon drug withdrawal. While reverting to LSC state is one established mechanism of chemotherapy tolerance in AML, the mechanism of tolerance/persistence in response to FLT3 inhibition is less understood.

### The role of drug-induced gene expression changes

A global understanding of the initial response to drug treatment can delineate the mechanism by which DTPs survive and eventually lead to resistance. A recent study by Melgar et al. [109] conducted an unbiased whole-genome transcriptome analysis as well as a peptide phosphorylation profiling of FLT3 mutant cells before and after treatment with quizartinib for 6 or 12 h. They reported that FLT3 inhibition triggers an immediate global change in gene expression and highlighted that innate immune and inflammatory pathways were significantly upregulated after treatment. Similarly, our group observed a significant upregulation of inflammatory pathways following FLT3 inhibition for 48 h in FLT3 mutant cells, but not in WT-FLT3 cells [101]. Interestingly, the immediate gene expression changes observed were maintained even after a prolonged inhibition of FLT3 suggesting that they can play a role in the eventual acquisition of resistance-conferring mutations.

Targeted kinase inhibitor (TKI)-induced upregulation of immune pathways has also been observed in various cancer models [110–112]. For example, treatment of EGFR mutant lung cancer cells with EGFR-targeted inhibitors resulted in inflammation mediated by cytokines, chemokines, type 1 IFN as well as recruitment of innate immune cells [111]. This drug-induced inflammation has been implicated to be the cause of acneiform rash, a skin inflammation, a common side effect of EGFR inhibitors observed in 49–95% of treated patients [110]. Similar drug-induced inflammation has also been observed in other types of cancers treated with drugs targeted at various kinases, such as BRAF, ALK, and c-MET [110, 113]. Taken together, inhibition of FLT3 as wells as other RTKs induces inflammation through the innate immune pathways. An important question that remains to be elucidated is the mechanism by which FLT3 inhibition induces the innate immune response pathways.

Drug-induced stress and cell death cause cells to release damage-associated molecular patterns (DAMPs), such as host nonnuclear/mitochondrial DNA or RNA, HMGB1, heat shock proteins etc., which have been shown to trigger “sterile inflammation” [114]. Thus, it is possible that DAMPs released by dying or stressed cells can trigger the innate immune response and inflammation. On the other hand, FLT3 inhibition has been shown to induce cell death through the apoptotic pathway which is nonimmunogenic. Furthermore, the study by Melgar et al. [109] showed that inflammation can be induced in cells treated with low-dose FLT3 inhibitor for a short period of time (6 and 12 h), which is enough to inhibit FLT3 but not induce cell death. These observations suggest that induction of inflammation is specific to inhibition of FLT3 or other relevant RTKs. Therefore, it is possible that inhibition of FLT3 can induce a direct or indirect interaction of FLT3 or downstream targets with inflammatory regulators to induce inflammation.

Since FLT3 and other RTKs such as EGFR, BRAF, c-MET, and ALK share downstream signaling pathways, it is possible that one or more of the downstream signaling proteins directly or indirectly affect inflammatory pathways. For instance, a study showed that activation of the PI3K pathway along with treatment with LPS or other toll-like receptor (TLR) agonists promotes the production of anti-inflammatory cytokines while reducing pro-inflammatory cytokines through Akt’s inhibitory action on GSK3 [115]. However, inhibition of Akt or other PI3K pathway proteins, which results in dephosphorylation and activation of GSK3, along with TLR activation resulted in increased production of pro-inflammatory cytokines, such as IL-6, IL-1β, TNF-α, and IFN-γ while decreasing the anti-inflammatory cytokine IL-10 [115]. They demonstrated that GSK3 mediates the association of p65 subunit of NF-κB with the nuclear coactivator CBP by negatively regulating CREB, which competes with p65 NF-κB for binding CBP [115]. Hence, it is possible that inhibition of the PI3K pathway by TKIs, including FLT3 inhibitors, contributes to inflammation through GSK3-induced augmentation of NF-κB activity. On the other hand, the MAPK pathway has been shown to get reactivated following a brief inhibition in FLT3 inhibitor treated cells [101, 109]. Hence, the MAPK pathway could also fuel inflammation in addition to promoting cell survival.

Collectively, therapy-induced inflammation is an important mechanism of drug tolerance and cell survival. Our group has demonstrated that anti-inflammatory glucocorticoids synergize with FLT3 inhibitors in inducing a more complete cell death and decreasing DTPs. Inhibition of IRAK1/4, NF-κB as well as other inflammatory pathways has also been shown to augment cell death induced by FLT3 inhibition [109, 116]. This highlights the importance of understanding and targeting drug-induced cellular stress response as a feasible strategy to prevent MRD and possibly relapse and resistance.

## Conclusion

The recent approval of two FLT3-targeted drugs for the treatment of FLT3 mutant AML patients is a significant advancement toward a better survival rate for a patient population that has a poor prognosis. However, the lack of durable remission in patients treated with single-agent FLT3-targeted therapies blunts their benefit and highlights the need for a continued effort to improve treatment modalities. Receptor- and nonreceptor-related mutations, epigenetic changes, and signaling pathway alterations that are preexistent or acquired could all be at play in driving FLT3 inhibitor resistance. It is crucial to identify and preemptively target these alterations early at the MRD stage in order to prevent relapse and improve survival.

## Data Availability

The datasets supporting the conclusions of this review article were generated by other research groups as well as our group and are listed in the reference section.

## Abbreviations

A-Loop: Activation loop
AML: Acute myeloid leukemia
AP-1: Activating protein-1
BM: Bone marrow microenvironment
CBFβ: Core binding factor beta
CR: Complete remission
CRc: Composite complete remission
CREBP: Cyclic AMP response element binding protein
CXCL12: C-X-C motif chemokine 12
DAMP: Danger-associated molecular pattern
DNMT3A: DNA methyl transferase 3A
DSB: Double-stranded break
DTP: Drug-tolerant persister
DUSP: Dual-specificity phosphatase
EGFR: Epidermal growth factor receptor
ERK: Extracellular-signal-regulated kinase
FGF2: Fibroblast growth factor
FL: Flt3 ligand
GRB2: Growth factor receptor-bound protein 2
GSK3: Glycogen synthase kinase 3
HCT: Hematopoietic stem cell transplant
IDH: Isocitrate dehydrogenase
ITD: Internal tandem duplication
JM: Juxtamembrane
M-CSF: Macrophage colony-stimulating factor
MAPK: Mitogen-activated protein kinase
Mcl-1: Myeloid cell leukemia 1
MEK: Mitogen-activated protein kinase
MLL: Mixed lineage leukemia
MTD: Minimal-tolerated dose
NF-kB: Nuclear factor kappa-light-chain-enhancer of activated B cells
NHEJ: Nonhomologous end joining
NPM: Nucleophosmin
PDGFR: Platelet-derived growth factor
PI3K: Phosphoinositide 3-kinase
PML: Promyelocytic leukemia protein
RAR: Retinoic acid receptor
ROS: Reactive oxygen species
RTK: Receptor tyrosine kinase
RUNX: Runt-related transcription factor
SH2: Src Homology 2
SHC: SH2 domain-containing protein
SHIP: SH2 domain-containing inositol polyphosphate 5-phosphatase 1
SOS: Son of Sevenless
SPRY: Sprouty
STAT: Signal transducer and activator of transcription
TET2: Tet methylcytosine dioxygenase 2
TKD: Tyrosine kinase domain
TKI: Tyrosine kinase inhibitor
TLR: Toll-like receptor
WT-FLT3: Wild-type FLT3
mTOR: Mammalian target of rapamycin
